# Thanks to all those who reviewed for *Research Involvement and Engagement* in 2015

**DOI:** 10.1186/s40900-016-0017-z

**Published:** 2016-02-11

**Authors:** Richard Stephens, Sophie Staniszewska

**Affiliations:** 1 Involved and engaged patient, Stevenage, Herts, UK; 2 Royal College of Nursing Research Institute, University of Warwick, Coventry, England

## Abstract

A peer-reviewed journal would not survive without the generous time and insightful comments of the reviewers, whose efforts often go unrecognized. Although final decisions are always editorial, they are greatly facilitated by the deeper technical knowledge, scientific insights, understanding of social consequences, and passion that reviewers bring to our deliberations. For these reasons, the Editors-in-Chief and staff of *Research Involvement and Engagement* warmly thank the 82 reviewers whose comments helped to shape the journal, for their invaluable assistance with review of manuscripts Volume 1 (2015).

**Julia Abelson**

Canada

**Adewale Adebajo**

UK

**Christine Allmark**

UK

**Peter Beresford**

UK

**Helena Binder**

UK

**Kate Boddy**

UK

**Laura Bond**

UK

**Jonathan Boote**

UK

**Louca-Mai Brady**

UK

**Rhianna Broadway**

UK

**Rod Buchanan**

UK

**Helen Bulbeck**

UK

**Iain Chalmers**

UK

**Ruth Chandler**

UK

**Imogen Chappelow**

UK

**Paul Charlton**

UK

**Tina Coldham**

UK

**Sally Crowe**

UK

**Maarten de Wit**

Netherlands

**Simon Denegri**

UK

**Krysia Dziedzic**

UK

**Stuart Eglin**

UK

**Jim Elliott**

UK

**David Evans**

UK

**Karen Facey**

UK

**Sheila Fisher**

UK

**Alison Ford**

UK

**Jacqui Gath**

UK

**Andy Gibson**

UK

**David Gilbert**

UK

**Joanna Groves**

UK

**Lee Gunn**

UK

**Lynn Hampson**

UK

**Janet Harris**

UK

**Kirstie Haywood**

UK

**Karen Inns**

UK

**Kristin Liabo**

UK

**Eric Low**

UK

**Sarah Markham**

UK

**Rachel Matthews**

UK

**Anne McKenzie**

Australia

**Virginia Minogue**

UK

**Carole Mockford**

UK

**Kerstin Morrison**

UK

**Shahid Muhammad**

UK

**Terry Mumford**

UK

**Matt Murray**

UK

**Luis Nacul**

UK

**Mary Nettle**

UK

**Gill Norman**

UK

**Ruth Northway**

UK

**Jack Nunn**

Australia

**Sandy Oliver**

UK

**Michael Osborne**

UK

**Sophie Petit-Zeman**

UK

**Claire Planner**

UK

**Karen Poole**

UK

**Katrina Randle**

UK

**Janette Rawlinson**

UK

**Amanda Roberts**

UK

**Jan Rose**

UK

**Kate Seers**

UK

**Natalie Simon**

UK

**Ann Single**

Australia

**Mark Smith**

UK

**Rosamund Snow**

UK

**Sophie Söderholm Werkö**

Sweden

**Kristina Staley**

UK

**Roger Steele**

UK

**Jackie Street**

Australia

**Jackie Sturt**

UK

**Mark Sujan**

UK

**Maryrose Tarpey**

UK

**Stephanie Tierney**

UK

**Liz Tutton**

UK

**Janet Wale**

Australia

**Scott Weich**

UK

**Margaret Wilcox**

UK

**Tracey Williamson**

UK

**Patricia Wilson**

UK

**Roger Wilson**

UK

**Charles Zandoh**

Ghana

